# Patient with agammaglobulinemia produces anti-SARS-CoV-2 reactive T-cells after CoronaVac vaccine

**DOI:** 10.1016/j.clinsp.2022.100007

**Published:** 2022-02-02

**Authors:** Telma Miyuki Oshiro, Lais Teodoro da Silva, Marina Mazzilli Ortega, Sandro Felix Perazzio, Alberto Jose da Silva Duarte, Magda Carneiro-Sampaio

**Affiliations:** a Laboratório de Investigação Médica em Dermatologia e Imunodeficiências (LIM 56), Hospital das Clínicas da Faculdade de Medicina da Universidade de São Paulo (HCFMUSP), São Paulo, SP, Brazil; b Division of Rheumatology, Universidade Federal de São Paulo, São Paulo, SP, Brazil; c Laboratório Central, Hospital das Clínicas da Faculdade de Medicina da Universidade de São Paulo (HCFMUSP), São Paulo, SP, Brazil; d Instituto da Criança e Adolescente (ICr), Hospital das Clínicas da Faculdade de Medicina da Universidade de São Paulo (HCFMUSP), São Paulo, SP, Brazil

**Keywords:** COVID-19, Coronavirus Disease 2019, SARS-CoV-2, Severe Acute Respiratory Syndrome Coronavirus 2

Since the first case of COVID-19, at the end of 2019, SARS-CoV-2 has spread rapidly worlwide, causing the death of more than 5 million people to this date and profoundly impacting population lifestyles.

Clinical manifestations of COVID-19 occur over a wide spectrum of severities, ranging from asymptomatic, mild, moderate, severe, and critical cases, which can be fatal. This enormous variability might be due to specific personal features, including the individual immune response, which can determine disease clinical course and outcome. Therefore, failures/defects of the immune system, as in primary immunodeficiencies, should present a greater potential risk for more severe forms of COVID-19.

Primary immune deficiencies, currently called Inborn Errors of Immunity (IEI), are genetic disorders classically characterized by an increased susceptibility to infection caused by a disruption in the development or regulation of an immune pathway.^1^^,^^2^ X-Linked Agammaglobulinemia (XLA), a type of IEI also called Bruton's agammaglobulinemia, is a rare immunodeficiency disorder characterized by absent/very low mature B lymphocytes in blood and tissues.^3^ XLA is caused by *BTK* gene mutations, which encode the Bruton or B-cell Tyrosine Kinase (BTK) protein, and play pivotal roles in pro-and pre-B cell maturation. *BTK* loss-of-function mutation interferes with B cell development resulting in very low (or absent) levels of serum and mucosal immunoglobulins and high susceptibility to extracellular bacterial, entero-, and respiratory virus’ infections.^4^ In contrast, T-cells are normal in number and functions, with no change in their proliferative responses to antigens.^5^

Previous reports have demonstrated that XLA patients are at risk for severe disease with SARS-CoV-2 infection. Authors have reported clinical symptoms with important increase in inflammatory markers , associated with long viral persistence, suggesting a role for antibodies in reduction of viral load.^6^^,^^7^ Ponsford et al. (2021) observed that XLA patients remain susceptible to severe disease. Persistent infection is common and is likely to carry a significant risk of novel variant evolution.^6^^,^^7^

Vaccination is a safe and effective tool to induce a protective immune response in immunocompetent individuals. Immunocompromised patients, in turn, have an increased susceptibility to vaccine-preventable infections,^8^^,^^9^ emphasizing the importance of vaccination in this group whenever possible. Although in some IEI conditions, as in XLA, low or absent antibody response to vaccines is observed, vaccination may induce other protective immune mechanisms, such as the cellular immune response.^9^ Thus, the immune status of vaccinated immunodeficient individuals is crucial.

Currently, multiple anti-COVID-19 World Health Organization (WHO) approved vaccines are being used worldwide. These vaccines are either mRNA, replication-deficient vector, inactivated whole virus, or protein-based vaccine. The European Society for Primary Immune Deficiency (ESID) recommends that IEI patients receive COVID-19 vaccinations that are not live vaccines, but based on killed/inactivated viruses or on mRNA-based vaccines.^10^

In Brazil, the first anti-COVID-19 immunizer to be used on a large scale was the CoronaVac (SinoVac), a vaccine based on the inactivated whole virus. The entire inactivated virus provides the whole antigenic repertoire for the immune system, unlike other vaccines that are based only on the spike protein. Hypothetically, a more diverse antigenic repertoire can bring advantages against the appearance of viral variants, selected mutations of which usually appear in the spike protein.

Here, we describe the case of a 32-year-old male XLA patient, diagnosed in the second semester of life and has since undergone gamma globulin replacement therapy. He has regularly received immunoglobulin since the second semester of life, currently using the preparation from Green Cross Corp (South Korea) by the intravenous route. He has presented a benign evolution without any severe infections and is an engineer undertaking normal activities. The mutation, identified as c.608_610delCGCinsTGGTG (p. P203Lfs*13), is a 3 base-pair deletion in exon 8, resulting in a premature stop codon further downstream.

The patient received two doses of the anti-COVID-19 inactivated whole virus-based vaccine CoronaVac (Sinovac) 4 weeks apart, with no significant side reactions. Blood samples were obtained 3 weeks after the 2^nd^ dose to determine serological levels of anti-SARS-CoV-2 neutralizing and IgG antibodies directed to trimeric spike glycoprotein of SARS-CoV-2. The cellular immune response was assessed by IFN-γ intracellular expression in CD3 T lymphocytes, after stimulation with a peptide pool containing the immunodominant sequence of the spike, membrane, and nucleocapsid proteins of SARS-CoV-2.

As expected, no detectable levels of serological antibodies were observed. Conversely, IFN-γ production by T-lymphocytes was comparable to samples from 36 vaccinated age-matched healthy controls, who also received 2 CoronaVac doses, presenting the same median value as healthy individuals (Fig. 1).

**Fig. 1:**
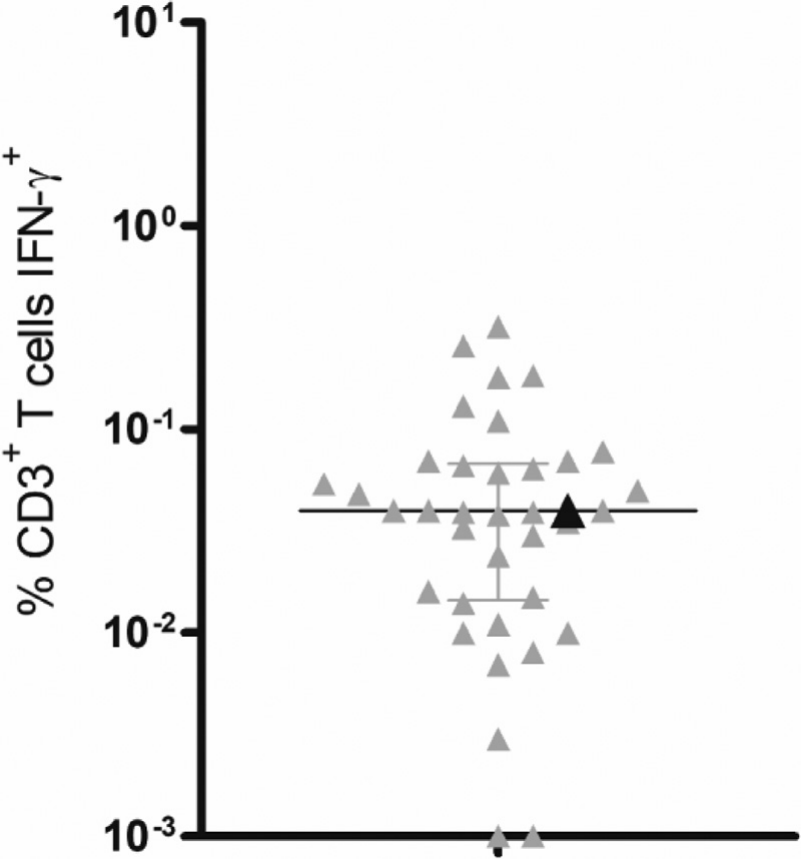
T-cell responses to SARS-CoV-2 peptide pools. PBMCs from CoronaVac vaccinated healthy donors (gray triangles) (n=36) and the XLA patient (black triangle) were incubated for 18h with a mixture of grouped SARS-CoV-2 peptide pools (membrane, nucleocapsid and spike) at a final concentration of 1 μg/mL. The logarithmic scale represents the percentage of CD3+ T-cells producing IFN-γ. Scatterplots show lines at the median with interquartile ranges. IFN-γ production was analyzed by intracellular flow cytometry.

Vaccine-induced immunity depends on a complex and multifaceted mechanism involving many immune components. However, single components, such as antibody responses, are often accepted as an immune correlate of protection, mainly because serologies are accessible and practical tests.^11^ In fact, as with most vaccines under current use, the immunizing agent's effectiveness has been commonly related to its ability to induce specific antibody production. Specific T-cell mediated immunity has rarely been assessed due to technical complexity and high costs

A recent publication showed evidence for neutralizing SARS-CoV-2 antibodies as protective correlates for COVID-19 vaccines.^12^ Conversely, serological tests for antibodies are not a precise indicator of the complexity and durability of immune memory to SARS-CoV-2 . In fact, sustained T-cell immunity, despite a decline in antibody response, was observed months after infection,^13^^,^^14^ suggesting that other immune components can contribute to protective immunity. Moreover, a robust T-cell response was observed in IEI patients after infection with SARS-CoV-2,^15^ showing the contribution of other immune compartments in the protective response. Together, these findings emphasize the need for cellular response assessment in individuals with antibody deficiencies for a better understanding of the anti-SARS-CoV-2 viral or vaccine-induced immune response.

Some studies have shown that mRNA-based vaccines are able to stimulate a cellular response in IEI patients, although the therapies being used and gene defects can affect vaccine immunogenicity.^16^ Interestingly, particularly for XLA patients, immunization with the mRNA-based vaccine BNT162b2 (Pfizer Biontech) resulted in a robust T-cell response comparable to healthy donors.^17^^,^^18^

This case report emphasizes the relevance of immunizing patients with antibody production disorders. To our knowledge, this report is the first description of cellular response in an XLA patient immunized with CoronaVac.

## Conflicts of interest

The authors declare no conflicts of interest.
